# Is Better Knowledge about Health Benefits of Dietary Fiber Related to Food Labels Reading Habits? A Croatian Overview

**DOI:** 10.3390/foods11152347

**Published:** 2022-08-05

**Authors:** Marija Ljubičić, Marijana Matek Sarić, Ivana Rumbak, Irena Colić Barić, Ana Sarić, Draženka Komes, Zvonimir Šatalić, Boris Dželalija, Raquel P. F. Guiné

**Affiliations:** 1 Department of Health Studies, University of Zadar, Splitska 1, 23000 Zadar, Croatia; 2 Faculty of Food Technology and Biotechnology, University of Zagreb, Pierottijeva 6, 10000 Zagreb, Croatia; 3 School of Medicine, Chatolic University of Croatia, Ilica 242, 10000 Zagreb, Croatia; 4 CI&DETS, Polytechnic Institute of Viseu, Av. José Maria Vale de Andrade, 3504-510 Viseu, Portugal

**Keywords:** food labels reading, dietary fiber, knowledge, consumption, health benefits

## Abstract

The aim of this cross-sectional study was to determine the associations between health dietary patterns, knowledge, and consumption of dietary fiber (DF) with frequency of food label reading on food products with special reference to DF. The study was conducted in 2536 Croatian adults using an original questionnaire. Multiple linear regression models were used to assess associations between food label reading habits and predictor variables. Our study confirms the association between habits regarding the reading of labels on food products, especially in relation to information about DF with the sociodemographic factors of respondents, dietary food patterns and DF consumption, as well as knowledge and sources of information about DF. Women, individuals with a university-level education, and those living in an urban environment had more frequent labels used. Food habits as well as eating outside of the home were positive predictors while eating fast food was a negative predictor of food label reading. Knowledge about DF, especially about its health benefits, was also associated with food label reading. The interpretation of associations could help with the design of effective public health programs. Targeted education campaigns to educate and sensitize the population about food labeling and monitoring may improve general knowledge about healthy food and its benefits, which include indirect effects on the prevention of non-communicable chronic diseases.

## 1. Introduction

The prevalence of nutrition-related health problems, such as chronic non-communicable diseases, has been progressively increasing [1,2]. It is estimated that, by 2030, 52 million people will be dying from these diseases each year [3]. Achieving optimal health for the population and target groups is a key aspect of public health programs aimed at achieving the Millennium Development Goals [4,5]. In achieving these goals, the promotion of healthy lifestyles and nutrition practices are two of the most challenging goals [2]. In order for people to realize the importance of a healthy and balanced diet with qualitatively and quantitatively balanced intakes of macro and micronutrients, raising knowledge in this area is important and is needed constantly. For example, it is well known that dietary fiber (DF) has a positive impact on health and the prevention of chronic diseases [6]. The consumption of DF is associated with many health benefits and contributes to the maintenance of good human health. Previous studies have confirmed that the consumption of DF is associated with lower cholesterol levels; the prevention of heart disease; the lowering and stabilization of blood glucose levels; and the prevention of obesity, diabetes, constipation, diverticulosis, and colon cancer, with some studies also reporting an impact on the prevention of breast cancer [7,8,9,10,11,12]. However, knowledge about DF sources in products and possible effects on health are lacking in the general population [13,14,15,16]. Additionally, studies have shown a certain level of disparity between knowledge and attitude towards the intake of DF. This means that people are aware of the role of fiber-rich foods in health conditions, but consumption of these foods is low [17]. These behaviors may limit the health benefits of DF [17]. On the other hand, many previous studies have confirmed the relationships among consumer awareness, knowledge, and the frequency of consumption of foods containing DF [13,14,16,18,19,20,21,22,23]. DF may be obtained from different dietary sources. However, DF representation varies from food to food. Studies have confirmed that whole grains, fruits, and vegetables are the richest DF sources. For example, whole cereals are an important source of DF, representing about 50% of the total DF intake. Vegetables represent about 30–49%, while fruits represent about 16% of the daily DF intake [24]. Despite this, the consumption of these dietary-rich foods is below the recommended level [19].

One possible way to improve this is to improve the knowledge of consumers about the consumption of these foods through the reading of food labels. Nutrition labeling literature suggests that the effectiveness of food labels is influenced by the consumers’ motivations, health or diet goals, nutritional knowledge, and time pressure. The available nutrition labeling literature details the dual-process theory that exists in food decision making. This depends on the consumer’s ability to quickly and automatically or slowly and deliberately choose food products based on the information on food labels [25]. Additionally, the hierarchy of effects model emphasizes the fact that consumers should be exposed to food labels and be able to understand them and that the ability to do this will be affected by consumers’ knowledge about nutrition [26]. Labels are important to consumers who are aware of the health benefits of certain food compounds, such as DF, and have enough knowledge about them. In addition to the fact that reading declarations may be determined by these personal characteristics and prior knowledge, it is very important to have distinct and convenient information on the food label [27].

Food labels provide information about the food product, such as its energy value, the contents of certain nutrients it contains (fats, saturated fatty acids, carbohydrates, sugars, proteins, salts, vitamins, minerals, DF, and other nutrients), and some mandatory components [28,29,30,31]. Therefore, food labels carry useful information about products and can be thought of as the identity card of a food product. They give the consumer the opportunity to consciously choose what to buy and allow them to make a final decision [28,30]. A potential problem is that legal regulation does not oblige the labeling of raw whole cereals, fruits, or vegetables, which are actually the richest categories, while all products made from these ingredients must contain a label [32]. Nevertheless, consumers who have better knowledge about fruits and vegetables or whole cereals as good sources of DF and their health benefit may be more inclined to choose and consume such foods or their products [19]. Additionally, studies confirm that higher scores for understanding labels were associated with consuming more vegetables [33]. Also, people who never sought information on health reported a significantly lower intake of fruit and vegetables, which is strongly linked to all of the major lifestyle diseases [23,34]. Knowledge about this potentially plays an important role in dietary behavior [35]. Less likely people read food labels if they have weaker knowledge and do not consume such healthy food. According to that, the knowledge and frequency of consumption of these rich dietary food products may be useful indicators in the analysis of food label reading. Additionally, sociodemographic characteristics and food habits may be associated with consumers’ habits of food labels [33,35].

Food labels may contain nutritional and health claims, which are two very important types of information [30]. A nutritional claim is any claim that states, suggests, or implies that a food has particular beneficial nutritional properties, e.g., low/”light-lite”/reduced content of fats, saturated fats, sugar, or sodium; high contents of DF, protein, vitamins, or unsaturated (monounsaturated or polyunsaturated fat) fats; or naturality [36,37]. A nutritional claim is only permitted if it is listed in the Annex of Regulation (EC) No 1924/2006, lastly amended by Regulation (EU) No 1047/2012 [37]. A health claim states, suggests, or implies that there is a link between a precisely determined food or precisely determined food ingredients and health [30]. In food labeling, health claims are strongly reviewed by organizations like the Food and Drug Administration (FDA) and can be put on specific food products to show that this food or food component may reduce the risk of a particular disease or could help with certain health-related conditions.

Nutritional and health claims are not mandatory on food products, and legal norms strictly prohibit the use of information that could mislead the purchaser or that attributes medicinal properties to food [29,37,38]. However, the growing link between diet and health has prompted the need for a clearer emphasis on nutrition labeling [6,39] in terms of highlighting nutritional and health benefits. According to the World Health Organization (WHO), the Food and Agriculture Administration (FAO), and the Codex Committee on Food Labeling, all food products should be marked in accordance with legal norms and in the language of the country in which they are offered to consumers [30,40]. Food labelling, consumer awareness, and comprehension of label information are crucial for determining, maintaining, and communicating food value [41]. It is also recommended that DF, due to its positive health effect, should be included in the supplemented mandatory food nutrition label [41]. Studies have confirmed that the use of food nutrition labels is associated with lower fat intake and belief of an association between diet and diseases [20]. Subjective norms and dietary health concerns are associated with the intention to use food labels [42].

Despite this, the reading of food labels is not widely carried out. In order to prevent chronic diseases related to unbalanced and unhealthy eating habits, public health organizations need to strongly support the development of healthy lifestyle habits, including healthy dietary patterns and the use of food-labels to promote the choice of healthier products [2]. This is important because food labels can assist consumers in the appraisal of a product. Furthermore, they could encourage the producer to enhance and adapt their manufacturing systems to meet their own expectations and needs for maintaining health and preventing chronic diseases. Consumers are very interested in how their diet can impact their health, prevent diseases, or aid in the management of diseases that they are already suffering from [6,29]. These factors may play crucial roles when making decisions on nutrition consumption. Scientists who emphasize the importance of labeling on consumers’ selections and future behaviors consider there to be links among choosing and consuming healthy foods, having knowledge about the relationship between diet and diseases, and improving eating habits through the reviewing and reading of food labels [2,28,40,43,44,45,46].

Despite these factors, little is known about associations between general and health knowledge about DF and food label reading. By assessing the knowledge of consumers about the health benefits of DF, the frequency of DF consumption, and the frequency of reading declarations on foodstuffs, it will be possible to assess the need for public health program activities that can act to improve health in a very simple way. Therefore, well-designed assessment studies are necessary to clarify the associations between knowledge about the benefits of DF and consumers’ awareness about the importance of reading food labels. Additionally, the lack of evidence on label reading and its association with consumer knowledge warrants future studies. Our assumption is that consumers do not pay enough attention to food labels, especially not to the extent that can change behaviors and thus affect health. The reason for this may be a lack of knowledge on this topic. Consequently, we assume that the lack of knowledge about DF is associated with a lower frequency of reading food labels about it. Our hypothesis is that an association exists between the reading of food labels, the frequency of DF consumption, food habits, and understanding the health benefits associated with DF consumption. If our assumptions are accurate, this study will directly confirm the necessary implementation of public health activities, which may improve knowledge and awareness among consumers about foods that are rich sources of DF, the reading of food labels, and the need to change negative food habits as well as other lifestyle habits. This may be crucial to prevent numerous chronic diseases and to preserve the population’s health.

The aim of this study was to determine the associations between sociodemographic factors, sources of information, and acquired knowledge, and the frequency of reading labels on food products with special reference to DF.

## 2. Materials and Methods

This descriptive study was based on a cross-sectional questionnaire, which was conducted on a non-probabilistic convenience sample of 2536 respondents from all counties of the Republic of Croatia, although participants from Dalmatia predominated. Ethical approval was granted by the Human Research Ethics Committee of Zadar General Hospital (No. 01-178-3/15). The entire data collection and analysis were conducted in accordance with the ethical standards of the Declaration of Helsinki.

### 2.1. Study Population

We recruited respondents from the general population by word-of-mouth; advertisements; and contact in universities, shopping centers, and downtown areas. This ensured wide demographic and socio-economic distributions. After signing the consent form, the respondents started filling in the questionnaire using the paper and pencil method, and each respondent was given privacy while filling out the questionnaire. The inclusion criteria were adult volunteers (i.e., 18 years or older) who fully completed the questionnaire and were taken into consideration, while respecting protocols regarding their informed consent and anonymity. The exclusion criteria were respondents who did not complete the entire questionnaire, tourists, and students from other countries.

### 2.2. Methods

The original questionnaire was written and validated in English by the CI&DETS Research Center of the Polytechnic Institute in Viseu, Portugal [47]. This English survey was translated into Croatian by two native Croatian speakers with experience in public health and nutrition studies. No questions were modified, removed, or added during the translation. In this survey, respondents were asked about demographic characteristics, knowledge, and sources of information about DF in foods or its importance for human health. They were also asked about habits regarding the reading of food labels and their awareness of concepts, definitions, and health effects of nutritional information with special reference to DF.

In the analysis of sources of information about DF, health institutions (health centers and hospitals), educational institutions (schools), media (radio, television, Internet), and educational materials (magazines, books) were taken into account. The entry of ordinal values from 1 to 6 was used to estimate how often sources of information about nutrition and DF are used (health centers and hospitals, radio, television, school, magazine and books, internet). Respondents ordered these sources from most important (1st) to least important (6th).

To assess the knowledge about DF, the questions were grouped into three categories: general knowledge about food fiber (6 items), specific knowledge about the relationship between DF and food (6 items), and health knowledge concerning the relationship between DF and health benefits related to the consumption of DF and the prevention of chronic diseases, such as cardiovascular diseases, hypercholesterolemia, colon cancer, breast cancer, and diabetes (10 items). The items were rated on a Likert scale from 1 to 5, where 1—completely disagree; 2—disagree; 3—neither disagree nor agree; 4–agree; and 5—completely agree. Responses to questions formulated in a negative way were scored using the same 5-point scale in reverse, so that higher scores always corresponded to greater knowledge. We summed the 6 items used to assess general knowledge and the 6 items used to assess specific knowledge using the minimum (1—never) answer to all questions and the maximal score (5—always) for all questions and for general and specific knowledge separately. For health knowledge, we summed each of the 10 items using the minimum score (1—never) and the maximal response (5—always) to all questions. Potential scores for general and specific knowledge ranged from 6 to 30, while the score range for health knowledge was 10 to 50.

To estimate the frequency of reading food labels, the Likert rating scale was used. This allows answers on a scale from 1 to 5 (1—never; 2—rare; 3—sometimes; 4—often; 5—always). The questions were grouped into two categories: reading labels about food products (2 items, with potential score of 2–10) and reading information on labels about DF (3 items, with potential score of 3–15). Overall label reading was computed as the sum of all answers on the food label scale (sum of all 5 items; the potential range of the summed score was 5–25). The minimum score of 5 points meant that respondents never read food labels, and the maximum score of 25 points meant that respondents always read food labels.

The Cronbach’s alpha scores, α = 0.77 for knowledge and α = 0.86 for reading food labels, were acceptable.

### 2.3. Statistical Analysis

The data were processed using SPSS 26.0 (IBM, New York, NY, USA). We used the Kolmogorov–Smirnov test to examine the normality of the distribution. As data were asymmetrically distributed, in the presentation of descriptive statistics, the median and interquartile range were used. The Mann–Whitney U test (for two variables) and the Kruskal–Wallis test (for comparisons of three or more variables) were used to examine the differences between variables. Linear regression was used to identify predictors affecting the prediction of food label reading or DF content checking on products as a consequence. The regression model included the following predictor variables: gender, level of education, living environment, sources of information, levels of general and specific knowledge about DF, and knowledge about health benefits. The reliability of the questionnaire was evaluated by the Cronbach alpha coefficient. In the mentioned statistical analysis, *p* < 0.05 was considered statistically significant.

## 3. Results

### 3.1. Sociodemographic Characteristics and Consumption of Fiber-Rich Foods in the Study Group

The average age of respondents was 30 (Mdn = 3.0; IQR = 24). The dominant group in the sample was females and younger respondents who were mostly living in urban areas and who had a university-level education (Table 1). The characteristics of the study population (age group, gender, living environment and education) are presented in Table 1. On average, the respondents stated that they consumed seven pieces of fruit per week (Mdn *=* 7; IQR *=* 6), corresponding to only one piece per day. The same results were found for weekly vegetable consumption (Mdn *=* 7; IQR *=* 4). Respondents stated that they consume whole cereals only twice per week (Mdn *=* 2; IQR *=* 4). Respondents stated that they eat outside the home twice per week (Mdn *=* 2.0; IQR *=* 5), and they eat fast food once per week (Mdn *=* 1; IQR *=* 1; Table 1).

### 3.2. Level of Knowledge and Sources of Information about DF

In the range from complete knowledge to complete ignorance, the level of knowledge about DF was Mdn *=* 17 (IQR *=* 4) for general knowledge about food fiber, Mdn *=* 18 (IQR *=* 5) for specific knowledge about the relationship between DF and food, and Mdn *=* 35 (IQR *=* 7) for health knowledge regarding the relationship between DF and health benefits in terms of the consumption of DF and the prevention of chronic diseases, (Table 1).

In the analysis of sources of information used for the acquisition of knowledge on DF, the results show that the internet is the primary source for respondents (45.6%), followed by magazine and books (15.2%), school (13.0%), television (13.7%), health centers and hospitals (12.8%), and then radio (7.5%) (Table 1).

### 3.3. Frequency of Reading Labels on Food Products

The average score for reading food labels was between rarely (Mdn *=* 2; IQR *=* 2) and sometimes (Mdn *=* 3; IQR *=* 2). The average frequency of reading labels about food products was Mdn *=* 6.0 (IQR *=* 4.0). The score for reading information about DF on labels was 7.0 (IQR *=* 5.0), while that for overall label reading was 14.0 (IQR *=* 7.0) (Table 1). Women, older respondents, participants from urban areas, and those with a university-level education were found to read food labels more frequently (Table 2). The frequency of reading food labels according to sociodemographic factors is shown in Table S1.

### 3.4. Associations among Reading Food Labels, Knowledge of DF, and Sociodemographic Characteristics in the Study Group

The multivariate linear regression confirmed the associations between reading food product labels, reading information on labels about DF, and overall label reading and sociodemographic characteristics, dietary food consumption, food habits, and knowledge about DF (Table 3). Female gender was associated with reading food product labels (β *=* 0.12, t *=* 6.31, *p* < 0.001), reading information on labels about DF (β *=* 0.11, t *=* 5.55, *p* < 0.001), and overall label reading (β *=* 0.13, t *=* 6.61, *p* < 0.001). Having a university-level education (*p =* 0.002; *p =* 0.008; *p =* 0.002) was a positive predictor for reading labels in general, while living in an urban environment was associated with reading food product labels (*p =* 0.023) (Table 3).

The frequency of dietary food consumption was also a positive predictor of label reading. For example, the frequency of whole cereal consumption was associated with reading food product labels (β *=* 0.21, t *=* 10.54, *p* < 0.001), reading information on labels about DF (β *=* 0.21, t *=* 10.16 *p* < 0.001), and overall label reading (β *=* 0.23, t *=* 11.48, *p* < 0.001). On the other hand, the frequency of fruit consumption was also associated with reading food product labels (*p* < 0.001) and overall label reading (*p =* 0.014), but there was no association with reading information on labels about DF (*p =* 0.233) (Table 3).

Eating outside of the home was only positively associated with reading labels on food products (β *=* 0.05, t *=* 2.49, *p =* 0.013), while eating fast food was negatively associated with reading labels on food products (β *=* −0.13, t *=* −6.16, *p* < 0.001), reading information on labels about DF (β *=* −0.07, t *=* −3.10, *p =* 0.002), and overall label reading (β *=* −0.11, t = −4.94, *p* < 0.001) (Table 3).

We did not find an association between reading food labels and the primary source of information about DF. There was a negative association when the source was magazines or books, but this association was weak (β *=* −0.05, t *=* −2.41; *p =* 0.016 for reading information on labels about DF; and β *=* −0.04, t *=* −2.08, *p =* 0.038 for overall label reading) (Table 3).

General knowledge about food fiber, specific knowledge about the relationship between DF and food, and knowledge about the health benefits of DF were associated with all categories of label reading. We found that knowledge about the health benefits of DF was more highly associated with reading information on labels about DF than other types of knowledge (β *=* 0.16; t *=* 8.41; *p* < 0.001), (Table 3).

## 4. Discussion

The aim of this cross-sectional study was to assess associations between food label reading, the consumption of food rich in DF, the general eating patterns of the examined population, and knowledge about DF. This study shows that the reading and analysis of food labels in the general population are associated with general and specific knowledge about DF as well as knowledge about the relationship between DF and health.

In our study, we found that general knowledge about DF, specific knowledge about the relationship between DF and food items, and knowledge about the health benefits of DF were associated with reading labels on food products, reading information about DF, and overall label reading. In addition, in our study, we found positive associations between the frequency of dietary food consumption and label reading, with special emphasis on whole cereals. Similar results were mentioned in other studies [15,46,48]. It is possible that people who have positive eating habits are more likely to read food labels. Additionally, it is possible that extensive promotion of healthy foods and greater knowledge increases a person’s awareness about healthy nutrition with special reference to DF. This may have a positive impact by promoting a healthy lifestyle [15,49,50]. Despite these factors, it appears that the typical Croatian consumer is unconcerned about the DF data on labels. It seems they have a low level of interest in food labels and they read food labels only rarely or sometimes. A Portuguese study about attitudes towards food labels presented similar results, showing that Portuguese consumers also have a low frequency of food label reading [47]. Similar results were found in many other studies [41,42,51].

In our study, the frequency of dietary food consumption was also a positive predictor of label reading. For example, the frequency of consumption of whole cereals was associated with food label reading, reading information about DF, and overall label reading. It is possible that respondents who consume DF-rich foods more often read declarations more frequently, because they have a greater need to look for the presence of DF. Consequently, they are more inclined to buy foods that are richer in DF. It is also possible that those who frequently consume foods rich in DF take more care of their own health, and therefore their knowledge about it is greater. Despite the statistically significant association, the relationship between reading labels and the consumption of food items rich in DF is relatively weak, which may indicate that the consumption of DF is below the recommended level. Despite the recommended daily consumption levels being three to five pieces of fruit, three meals containing vegetables, and three meals containing whole cereals, studies have shown insufficient consumption of DF, and this was confirmed in our study. An increase in knowledge about the health benefits of DF and improved labeling and reading about the presence of DF in food could contribute to better consumption of DF in food. In our study, the level of knowledge about DF was found to be unsatisfactory, and we suggest that this may have a negative effect on the reading of food labels and or asking for information about DF on food products.

Eating outside the home is often a key component of a modern and fast-paced lifestyle and includes eating “on the go“ [52]. This depends on personal attitudes and usual eating habits. Some studies have confirmed that a high rate of eating outside the home is related to a poorer diet quality; a higher intake of energy, sugar, and fats; and a lower intake of fiber, fruit, and vegetables [53]. Additionally, studies have confirmed that eating outside the home is occurring more frequently due to lifestyle changes, but the effect of this may vary depending on the country and eating out is not always necessarily unhealthy. In some countries, this may be an indicator of a higher socioeconomic status, while in other countries, this is a cheaper way of eating, usually marked by frequent fast food consumption and poor lifestyle habits [54]. For example, in our study, reading labels on food products showed a weak positive association with eating outside the home. On the other hand, the negative associations between eating fast food and reading food labels may indicate that fast food consumption may negatively affect the reading of food labels, neglecting their significance.

Many studies have shown that reading labels may be affected by age, gender, marital status, socioeconomic status, education level, living environment, food-related motivations, nutrition knowledge, lifestyle habits, special dietary needs, being concerned about health, and other factors [53]. The nutritional information on food labels is very rarely or only occasionally taken into account, and mostly, connectivity is achieved due to sociodemographic factors such as gender, level of education, and the living environment. Our results are similar to the results of other studies in which the frequency of label reading was also found to be significantly higher in women and highly educated respondents in urban areas. Other studies have also confirmed that women read food labels more frequently than men. The reason for this may be that women have a greater interest in healthy eating because of its impact on body satisfaction and self-image [55,56]. Some studies found that women report more negatively regarding their perceptions of unhealthy foods such as sugar, red meat, white flour, and additives, which suggests that women eat more healthily than men but also have more body shape concerns and food and health anxieties [57].

Our bivariate analysis showed that, when buying food products, middle-aged respondents usually consult the food labels and pay more attention to health characteristics and, thus, the DF content in foods. Younger people pay more attention to nutritional information in general. This differs from the results of Satia et al., who found that the older sector of the population uses nutritional labels more in general [58]. It can be assumed that the incidence of diseases gets higher with age, which may be why we found that the middle-aged respondents check the food labels and content of DF in products more often than the younger sector of the population. It is possible that middle-aged and elderly respondents pay more attention to the need for a diet rich in DF because of the higher risk of developing certain diseases in these age groups. However, this was not confirmed in our regression models, because the predictor of age was not associated with the reading of food labels. Additionally, disease risk factors and their prevalence were not examined in this study. However, due to the focus on disease prevention or the possible deterioration of their own health and the alleviation of existing symptoms of diseases, some consumers need a certain diet and must consume foods with a specific dietary composition.

Various reasons exist to explain why consumers use or do not use nutritional labels. These may include consumer perceptions of positive impacts on their own health, such as the prevention of or therapy for chronic diseases like diabetes, cardiovascular diseases, and obesity. However, variables may also include rising health literacy levels and motivational factors for future positive health behaviors [51,53,59,60,61]. In addition, Kim et al. found that nutrition label reading is associated with higher intakes of calcium and vitamin C, a lower intake of calories, and a higher energy ratio for protein intake [62]. Kessler and Wunderlich noted that people with diabetes read food label information more often than general consumers [59]. However, the same authors also noted that the use of nutritional labels has a limited effect on nutrition knowledge gain, although some studies have confirmed that the reading of nutritional labels is associated with a lower prevalence of metabolic syndrome and a lower fat intake [20,59,63]. Additionally, a longitudinal American study found that nutrition label users have a decreased risk of getting a diabetes diagnosis [60]. Our observations confirm that there is a correlation between the consumption of fruits, vegetables, salads, and whole cereals, and label reading. However, some studies have not found an association between label use and fruit and vegetable consumption or have confirmed that people are least likely to view labels on fruits and vegetables [20,64].

However, studies have confirmed that levels of knowledge and education, motivation for health behaviors, lifestyle, and environment can influence eating habits and food consumption [19,23,37,61]. Studies have also confirmed that having a university-level education is associated with having greater knowledge and paying more attention to food labels. It is possible that those with a higher level of education have a greater level of interest in obtaining new knowledge and having a healthy lifestyle [22]. Despite this, in parts of the living environment, the awareness of food labels is still debatable, as is their influence on consumers’ attitudes, preferences, and quality perceptions. Some studies have suggested a need for the strengthening of research base systems used on labels and their influences on consumer behaviors to obtain more valid and compliant evidence-based policies in this area [65,66]. Still, findings from prior research on the role of labels are particularly controversial, with some authors considering them a critical antecedent of consumers’ behavior and others finding them a factor of minor influence [41,53].

Although there are some differences in food labeling among individual countries, consumers generally consider food labels on products, particularly information related to claims, as beneficial for health maintenance or improvement. They could also be important for consumers’ analysis and judgmental systems and, ultimately, could affect consumers’ behavior and promote and satisfy socioeconomic objectives, such as improving human health and safety as well as environmental factors associated with food production and consumption [44,67]. Therefore, food information in terms of food labels, other than factors that legally have to be present on products, should be legible and scientifically updated in order to better educate and inform the consumer.

On the other hand, studies have confirmed that a significant proportion of consumers do not have sufficient knowledge to allow them to understand the values stated on food labels and do not clearly distinguish between nutrient content, structure function, and health or nutrition claims. Thus, there is a need to increase the level of consumer knowledge [44,45,67]. Consumer confidence is most often based on trust in certain brands, labels, and traditions regarding certain foods [22]. These results are in line with our knowledge that most consumers only review food labels sometimes. It is possible that they read food labels briefly or review only the origin and shelf life of the food and have a certain level of indecision and partial knowledge regarding the importance of reading food labels. This may be due to the time required to analyze data as well as knowledge on where the information collection and analysis took place. A number of studies have confirmed that, for consumers, it is best to keep product information short and use both sides of the package, for example, present short claims on the front and more detailed information on the back [44,68]. Clearly, consumers prefer short, concise information that creates a more convincing and positive picture without having to read a lot of text [44,68]. The reason for this is that information overload can occur due to exposure to too much information, especially at the point of purchase where consumers generally have limited time or are exposed to various distractors that can interfere with the analysis of read information [31,43,67]. However, although an excessive amount of information increases the risk of consumer overload, there is a growing need to provide more product information to promote the consumption of a balanced and healthy diet [31,43]. It is possible that the implementation of front-of-pack nutritional labels, like warning labels, could increase label reading and could warn consumers about critical nutrients that could represent a health risk, such as sugar, salt, or saturated fats [69].

The nutritional content information presented on labels has an effect on choice of food and stimulates the consumption of healthy food, but labels can also have an inadvertent effect by promoting excessive intake of certain nutrients or products [30]. Although having a food label on the product is mandatory, for some consumers, the presence of it leaves an impression that the food is healthy, and this “halo” effect may deter them from seeking further information about the food and its composition [44]. Additionally, food labels can create confusion if they are not presented in a way that is easily understood by consumers.

Even though consumers may be shopping with a health or diet goal in mind, particular distractions, such as time pressure, may have an impact on their attention to food labels and their nutrition or health claims. Consumers may not use nutrition labels if they are complicated and time-consuming [27]. Reading food labels may be related to different factors, for example, sociodemographic characteristics, food-related motivation, health orientation, point-of-sale, repeat purchase factors, and time and daily segments of food shopping [53,70]. Additionally, it is possible that respondents who shop in supermarkets and large shopping centers have more time to analyze food products when they wait in line compared with those who go to small shops. However, we did not examine the sales point at which they buy fiber-rich foods (supermarket, small shops), how long purchases last, or when consumers shop.

However, the health benefits of reading food labels are much greater than these findings, and this paper shows that there is a weak connection between consuming DF and reading food labels, with the largest share of impact being nutritional information and the analysis of the food label itself, rather than the DF itself. Additionally, in this study, we found a relatively weak correlation between general and health knowledge about DF and the frequency of reading labels. We did not find an association between sources of information about DF and label reading, despite other studies showing that nutritional knowledge provides support for food label use [45]. Accordingly, greater knowledge about the health benefits of DF is needed in the general population, because this may have a positive impact on the consumption of foods containing fiber, which may improve people’s health.

The strengths of this study are the relatively large sample size and the inclusion of questions on both general and health-related knowledge about DF and reading food product labels and information about DF on labels. Despite these factors, our results should be interpreted with the consideration of some limitations. First, this was a cross-sectional study, which means that it cannot conclude that there is a causal relationship between the use of nutritional labels and actual nutrient intake. Likewise, the labels for different food categories are quite different, so it is quite difficult to carry out comparisons, especially knowing that some raw produce with high DF do not need labels and that the variables of frequent consumption of whole grain, fruits, and vegetables are more like identifiers of the consumer food habits. Additionally, this study lacked data on the health statuses of respondents, possible bad habits in terms of smoking and physical activity, and the BMI data of respondents.

Hence, because food labels include information relating to both safety and nutritional content, they have been shown to be important for consumer protection [71]. Thus, it is very important that consumers are instructed to understand and have confidence in the information on food labels to allow them to correctly select and consume foods. Public health programs urgently need to raise public awareness about the importance of understanding nutritional labels to achieve healthy food consumption. Hence, emergency actions must be undertaken to increase attitudes towards the importance of food label reading, because it has been observed that many people do not pay attention to the nutritional information present on food labels that might help them with better food selections.

## 5. Conclusions

Monitoring dietary fiber on food labels was chosen as a topic, because it provides an instructive example for consumers in terms of understanding and reading different types of labels and claims known and distinguished by the law. This detailed analysis of the level of reading of nutritional labels and information about dietary fiber may help with the design of effective public health programs in Croatia and potentially other countries. Thus, targeted education campaigns should be developed to educate and sensitize the population about food labeling, monitor dietary fiber through food labels, and improve knowledge about healthy foods and their health benefits. The use of these very simple steps in public programs may increase and/or sensitize the population to consume healthy foods and be active in the prevention of chronic non-communicable diseases. Public health efforts should be made to help consumers to understand the nutritional information on food labels in order to carry out more effective decision making.

## Figures and Tables

**Table 1:** Sociodemographic characteristics, lifestyle, and knowledge about dietary fiber and frequency of food label reading in the study group (N = 2536).

Age (Years), Mdn (IQR)				30.0 (24.0)		
Age groups, N (%)						
under 25						1010 (39.9)
26–35						488 (19.3)
36–45						385 (15.2)
46–55						480 (19.0)
56–65						143 (5.7)
66 and older						24 (0.9)
Gender, N (%)						
Male						826 (32.6)
Female						1704 (67.4)
Education, N (%)						
Primary school						54 (2.1)
High school						1221 (48.3)
University degree						1250 (49.4)
Frequency of food consumption, Mdn (IQR)						
Fruit (pieces per week)						7.0 (6.0)
Salads (meals per week)						7.0 (4.0)
Whole cereals (meals per week)						2.0 (4.0)
Frequency of eating outside the home (times per week), Mdn (IQR)						2.0 (5.0)
Frequency of eating fast food (times per week), Mdn (IQR)						1.0 (1.0)
Sources of information about dietary fiber, N (%)						
	1st source	2nd source	3rd source	4th source	5th source	6th source
Health centers, hospitals	303 (12.8)	251 (10.6)	242 (10.2)	328 (13.8)	471 (19.9)	774 (32.7)
Radio	177 (7.5)	216 (9.1)	283 (12.0)	406 (17.1)	560 (23.6)	726 (30.7)
Television	331 (13.7)	467 (19.3)	552 (22.8)	533 (22.0)	342 (14.1)	196 (8.1)
School	306 (13.0)	314 (13.3)	451 (19.1)	487 (20.6)	391 (16.6)	411 (17.4)
Magazines, books	369 (15.2)	634 (26.2)	498 (20.5)	413 (17.0)	316 (13.0)	194 (8.0)
Internet	1120 (45.6)	384 (15.6)	246 (10.0)	215 (8.8)	173 (7.0)	317 (12.9)
Knowledge about DF, Mdn (IQR)						
General knowledge about food fiber						17.0 (4.0)
Specific knowledge about the relationship between dietary fiber and food						18.0 (5.0)
Knowledge about the health benefits of dietary fiber						35.0 (7.0)
Food label reading, Mdn (IQR)						
When I buy a food product, I usually consult the information on the label.						3.0 (2.0)
On the label, I usually look at the nutritional information related to the food.						3.0 (2.0)
In the nutritional table, I usually look at the fiber content of the food.						2.0 (2.0)
The amount of fiber is a factor to consider when choosing among similar foods.						3.0 (2.0)
If I buy a food that is referred to as “fiber rich” on the pack, I check the label to see the amount of fiber present.						2.0 (2.0)
Read labels about food products						6.0 (4.0)
Read information on labels about dietary fiber						7.0 (5.0)
Overall label reading						14.0 (7.0)

Note: Mdn (IQR)—Median (Interquartile range); N (%)—absolute number (percentage number); General knowledge of food fibers—variable range between 6 and 30; Specific knowledge about the relationship between dietary fiber and food—variable range between 6 and 30; Knowledge about the health benefits of dietary fiber—variable range is between 10 and 50; Items about food label reading were rated on a Likert scale from 1 to 5 (1—never; 2—rarely; 3—sometimes; 4—often; 5—always); Overall label reading—sum of all 5 items, variable range between 5 and 25.

**Table 2:** Food label reading habits presented by gender, living environment, education, and age for a sample of residents from Croatia (N = 2536).

	RFL 1 ^†^	RFL 2 ^†^	RFL 3 ^†^	RFL 4 ^†^	RFL 5 ^†^
Gender					
Female	1336.58	1349.94	1342.02	1319.50	1321.61
Male	1094.69	1064.42	1069.50	1123.60	1129.20
p *	<0.001	<0.001	<0.001	<0.001	<0.001
Living environment					
Rural	1122.82	1132.74	1210.12	1201.02	1255.90
Urban	1282.40	1278.24	1251.75	1257.50	1245.76
p *	<0.001	<0.001	0.208	0.092	0.763
Education					
Primary school	826.75	880.91	1066.12	1088.51	1089.66
Secondary school	1193.64	1174.69	1210.61	1178.01	1233.06
University degree	1334.27	1348.06	1298.29	1332.70	1286.38
p *	<0.001	<0.001	0.001	<0.001	0.037
Age (years)					
<25	1214.14	1311.75	1187.50	1260.93	1234.09
26–35	1230.75	1250.29	1262.04	1197.17	1216.12
36–45	1281.58	1223.20	1301.96	1266.81	1303.13
46–55	1356.99	1217.87	1328.78	1290.43	1296.33
56–65	1269.19	1126.65	1310.44	1293.59	1334.40
>66	1238.00	1155.39	1269.75	1100.91	1289.33
p *	0.013	0.019	0.003	0.284	0.182

* *p* < 0.05; Parameter estimates in each column are shown as the mean rank of the Mann–Whitney U test for gender and living environment or the mean rank of the Kruskal–Wallis test for education and age. ^†^ RFL *=* reading food labeling; RFL 1 = When I buy a food product, I usually consult the information on the label; RFL 2 = On the label, I usually look at the nutritional information relevant to the food; RFL 3 = On the nutritional table, I usually look at the fiber content of the food; RFL 4 = The amount of fiber is a factor to consider when choosing similar foods; RFL 5 = If I buy a food that referred to as having a “high fiber content“ or being ”fiber-rich” on the pack, I check on the label to see the amount of fiber present.

**Table 3:** Associations between lifestyle habits, knowledge about dietary fiber, and reading food labels in the study group determined using linear regression models.

	Reading Labels on Food Products			Reading Information on Labels about DF			Overall Label Reading		
	β	t	*p*	β	t	*p*	β	t	*p*
Sociodemographic characteristics									
Age	−0.04	−1.79	0.073	0.01	0.36	0.720	−0.01	−0.65	0.519
Women	0.12	6.31	<0.001	0.11	5.55	<0.001	0.13	6.61	<0.001
University-level education	0.06	3.08	0.002	0.05	2.65	0.008	0.06	3.12	0.002
Urban environment	0.04	2.28	0.023	−0.03	−1.78	0.075	0.00	−0.02	0.980
Dietary food consumption (frequency)									
Fruit (pieces per week)	0.07	3.62	<0.001	0.02	1.19	0.233	0.05	2.47	0.014
Salads (meals per week)	0.09	4.21	<0.001	0.08	3.70	<0.001	0.09	4.41	<0.001
Whole cereals (meals per week)	0.21	10.54	<0.001	0.21	10.16	<0.001	0.23	11.48	<0.001
Food habits									
Eating outside the home (times per week)	0.05	2.49	0.013	0.01	0.64	0.524	0.04	1.63	0.104
Eating fast food (times per week)	−0.13	−6.16	<0.001	−0.07	−3.10	0.002	−0.11	−4.94	<0.001
Source of information about DF (the least important)									
Health centers, hospitals	0.03	1.65	0.099	0.00	0.18	0.855	0.02	0.96	0.339
Radio	0.03	1.33	0.182	−0.01	−0.47	0.640	0.01	0.39	0.700
Television	0.02	1.33	0.185	0.01	0.30	0.765	0.02	0.85	0.398
School	−0.01	−0.66	0.512	−0.01	−0.71	0.475	−0.01	−0.74	0.460
Magazines, books	−0.02	−1.20	0.228	−0.05	−2.41	0.016	−0.04	−2.08	0.038
Internet	−0.03	−1.59	0.113	−0.04	−1.90	0.057	−0.04	−1.93	0.054
Knowledge about DF									
General knowledge about food fiber	0.09	3.83	<0.001	0.10	4.21	<0.001	0.11	4.48	<0.001
Specific knowledge about the relationship between DF and food	0.06	2.42	0.016	0.05	2.28	0.023	0.06	2.65	0.008
Knowledge about the health benefits of DF	0.09	4.62	<0.001	0.16	8.41	<0.001	0.14	7.51	<0.001

Note: DF—dietary fiber, β-beta standardized coefficient, t-statistic, *p*-*p* value.

## Data Availability

The data are available from the corresponding author.

## Supplementary Materials

The following supporting information can be downloaded at: https://www.mdpi.com/article/10.3390/foods11152347/s1, Table S1: Frequency of food label reading in accordance with sociodemographic factors of subjects; N (%).
