# Supplementary material for: Is Better Knowledge about Health Benefits of Dietary Fiber Related to Food Labels Reading Habits? A Croatian Overview
Source: Foods. 2022 Aug 5;11(15):2347. doi: 10.3390/foods11152347 (PMC9367754; doi:10.3390/foods11152347)
Supplement: Supplementary file 1 [file foods-11-02347-s001.zip › foods-1819164-SI.pdf]

foods MDPI

Supplementary Materials:

Supplemental Table S1. Frequency of food label reading in accordance with sociodemographic factors of subjects; N (%).

Variable	Scoring	Sex		Living environment		Education		
		Female	Male	Rural	Urban	Primary school	Secondary school	University degree
RFL 1†	Never	102 (6.0)	134 (16.4)	68(12.3%)	167(8.6)	12(22.2)	137 (11.3)	86 (6.9)
	Rarely	258 (15.2)	165 (20.2)	99 (17.9)	317 (16.3)	15 (27.8)	219 (18.1)	189 (15.2)
	Sometimes	472 (27.8)	211(25.8)	176 (31.8)	502 (25.9)	17 (31.5)	337(27.8)	326 (26.2)
	Frequently	566 (33.3)	198 (24.2)	148 (26.8)	608 (31.3)	6 (11.1)	341 (28.2)	417 (33.5)
	Always	300 (17.7)	109 (13.3)	62 (11.2)	346 (17.8)	4 (7.4)	177 (14.6)	227 (18.2)
	p*	<0.001		<0.001			<0.001	
RFL 2†	Never	217 (12.8)	226 (27.6)	124 (22.5)	317 (16.3)	21 (38.9)	250 (20.7)	171 (13.7)
	Rarely	355 (20.9)	204 (24.9)	127 (23.0)	425 (21.9)	13 (24.1)	282 (23.3)	263 (21.1)
	Sometimes	507 (29.9)	194 (23.7)	164 (29.7)	531 (27.4)	12 (22.2)	347 (28.7)	341 (27.4)
	Frequently	406 (24.0)	123 (15.0)	88 (15.9)	435 (22.4)	4 (7.4)	217 (18.0)	307 (24.6)
	Always	210 (12.4)	71 (8.7)	49 (8.9)	231(11.9)	4 (7.4)	112 9.3)	164 (13.2)
	p*	<0.001		<0.001			<0.001	
RFL 3†	Never	488 (28.8)	403 (49.5)	209 (37.9)	678 (35.1)	27 (50.0)	464(38.5)	399 (32.2)
	Rarely	540 (31.9)	199 (24.4)	160 (29.0)	571(29.6)	13 (24.1)	343 (28.4)	381 (30.7)
	Sometimes	464 (27.4)	143 (17.6)	130 (23.6)	472 (24.4)	9 (16.7)	282 (23.4)	316 (25.5)
	Frequently	144 (8.5)	53 (6.5)	35 (6.3)	158 (8.2)	4 (7.4)	87 (7.2)	105 (8.5)
	Always	56 (3.3)	16 (2.0)	18 (3.3)	53 (2.7)	1 (1.9)	30 (2.5)	40 (3.2)
	p*	<0.001		0.497			0.034	
RFL 4†	Never	178 (10.5)	171(20.9)	84 (15.2)	263 (13.6)	18 (34.0)	201(16.7)	129 (10.4)
	Rarely	219 (13.0)	121 (14.8)	88 (16.0)	250 (12.9)	6 (11.3)	188 (15.6)	146 (11.7)
	Sometimes	596 (35.3)	265 (32.3)	178 (32.3)	675 (34.8)	10 (18.9)	400 (33.1)	449 (36.1)
	Frequently	497 (29.4)	194 (23.7)	147 (26.7)	538 (27.8)	10 (18.9)	311 (25.8)	368 (29.6)
	Always	200 (11.8)	69 (8.4)	54 (9.8)	212 (10.9)	9 (17.0)	107 (8.9)	153 (12.3)
	p*	<0.001		0.254			<0.001	
RFL 5†	Never	473 (27.9)	341 (41.6)	169 (30.6)	639 (32.9%)	20 (37.7)	408 (33.7)	385 (30.9)
	Rarely	419 (24.7)	181 (22.1)	145 (26.3)	452 (23.3)	19 (35.8)	290 (23.9)	290 (23.3)
	Sometimes	418 (24.6)	154 (18.8)	125 (22.6)	437 (22.5)	6 (11.3)	275 (22.7)	289 (23.2)
	Frequently	250 (14.7)	91 (11.1)	67 (12.1)	271 (13.9)	4 (7.5)	147 (12.1)	189 (15.2)
	Always	138 (8.1)	52 (6.3)	46 (8.3)	144 (7.4)	4 (7.5)	92 (7.6)	94 (7.5)
	p*	<0.001		0.429			0.079	

*Chi-Square test; †RFL= reading food labeling; RFL 1 = When I buy a food product, I usually consult the label information; RFL 2 = On the label, I usually look at the nutritional information related to the food; RFL 3 = On the nutritional table, I usually look at the fiber content of the food; RFL 4 = The amount of fiber is a factor to consider when choosing among similar foods; RFL 5 = If I buy a food that is referred to as having a "high fiber content" or being "fiber rich" on the pack, I check the label to see the amount of fiber present.
